# Identification of deleterious rare variants in *MTTP*, *PNPLA3*, and *TM6SF2* in Japanese males and association studies with NAFLD

**DOI:** 10.1186/s12944-017-0570-y

**Published:** 2017-09-26

**Authors:** Supichaya Boonvisut, Ken Yoshida, Kazuhiro Nakayama, Kazuhisa Watanabe, Hiroshi Miyashita, Sadahiko Iwamoto

**Affiliations:** 1 Division of Human Genetics, Center for Molecular Medicine, Jichi Medical University, 3311-1 Yakushiji, Shimotsuke, Tochigi, 329-0498 Japan; 2 Jichi Medical University Health Care Center, Shimotsuke, Tochigi, 329-0498 Japan; 3 The Chulabhorn Graduate Institute, 54 Kamphangphet 6 Road, Laksi, Bangkok, 10210 Thailand

**Keywords:** Non-alcoholic fatty liver disease, Rare variants, Re-sequencing, Association study

## Abstract

**Background:**

Non-alcoholic fatty liver disease (NAFLD) is a disorder characterized by excessive fat deposits in hepatocytes without excessive alcohol intake. NAFLD is influenced by genetic factors, and the heritability has been estimated at 0.35 to 0.6 by twin studies. We explored rare variants in known NAFLD-associated genes to investigate whether these rare variants are involved in the susceptibility to NAFLD.

**Methods:**

The target genes for re-sequencing were *PNPLA3*, *TM6SF2*, and *MTTP*. All exons of these three genes were amplified from a discovery panel of 950 Japanese males, and the identified rare variants were further tested for genetic association in 3014 individuals from the Japanese general population and for in vitro functional evaluation.

**Results:**

Target re-sequencing analysis using next-generation sequencing identified 29 rare variants in 65 Japanese males (6.84%), 12 of which were newly identified base substitutions. A splicing mutation in *TM6SF2* that resulted in deletion of 31 amino acids was identified in an NAFLD case. Among eight genotyped rare single-nucleotide polymorphisms (SNPs; minor allele frequency < 0.02), rs143392071 (Tyr220Cys, *PNPLA3*) significantly increased (odds ratio = 3.52, *P* = 0.008) and rs756998920 (Val42Ile, *MTTP*) significantly decreased (odds ratio = 0.03, *P* = 0.019) the NAFLD risk. Functional assays showed that these two SNPs disrupted protein functions and supported the genetic association.

**Conclusion:**

Collectively, 1.79% of individuals in our studied population were estimated carriers of rare variants that are potentially associated with NAFLD.

**Electronic supplementary material:**

The online version of this article (10.1186/s12944-017-0570-y) contains supplementary material, which is available to authorized users.

## Background

Non-alcoholic fatty liver disease (NAFLD) is a disorder characterized by excessive fat deposits in hepatocytes in the absence of excessive alcohol intake. NAFLD shows a broad clinical spectrum including simple steatosis, non-alcoholic steatohepatitis, and cirrhosis [1]. In addition, NAFLD has emerged as an independent risk factor for cardiovascular diseases [2]. Although systemic adiposity is the principal risk factor for NAFLD, non-obese NAFLD and obese non-NAFLD are not rare phenotypes [3]. Ethnic and familial accumulation suggests that NAFLD is influenced by genetic factors, and the heritability has been estimated to be 0.35 to 0.6 by twin studies [4–6]. Genome-wide association studies (GWAS) have revealed multiple loci associated with NAFLD. Common variants near *neurocan-cartilage intermediate layer protein 2* region (*NCAN-CILP2*), *glucokinase regulator* (*GCKR*), *lysophospholipase like 1* (*LYPLAL1*), and *patatin like phospholipase domain containing 3* (*PNPLA3*) were identified in a GWAS of individuals of European descent [7]. Among these loci, *NCAN-CILP2* and *PNPLA3* have been repeatedly identified as being deeply associated with NAFLD in multiple ethnic groups [8, 9]. Recently, in the *NCAN-CILP2* region, a missense single-nucleotide polymorphism (SNP) in *transmembrane 6 superfamily member 2* (*TM6SF2*) (rs58542926) was identified as a functional variant in this region [10]. In *PNPLA3*, minor allele of a common missense SNP (rs738409) disrupts the enzymatic activity and enhances the susceptibility to NAFLD [11–13].

A GWAS of European individuals showed that the identified SNPs accounted for 4.4% of the variance in hepatic steatosis, but a family study showed NAFLD heritability of 0.27 [7]. This discrepancy, so called ‘missing heritability’, remains to be resolved. Heritability of an NAFLD-related trait, dyslipidemia, has been shown to be due partially to low frequency but highly effective variation [14]. Exome sequence analysis revealed the burden for deleterious rare variants in known dyslipidemia-associated genes and a newly identified gene [15]. However, a limited number of studies has reported NAFLD-associated rare variants [16], and no paper has shown the impact of rare variants on this common hepatic disease in the general population. The aim of this study was to explore rare variants in known NAFLD-associated genes and to investigate whether these rare variants are involved in susceptibility to the disease in the Japanese general population. In addition to *PNPLA3* and *TM6SF2*, *microsomal triglyceride transfer protein* (*MTTP*) was chosen as a target gene for re-sequencing. *MTTP* is the locus for abetalipoproteinemia, and patients with this disorder show severe hypocholesterolemia and hepatic steatosis. All exons of these three genes were amplified from a discovery panel of 950 Japanese males. The sample numbers of NAFLD and control cases were suitable for a case control study comparing females, and the numbers of subjects had sufficient prospective statistical power (0.8) even for rare variants (0.02 ≥ minor allele frequency ≥ 0.005) (Additional file 1: Table S1). The identified rare variants were further tested for genetic association in 3014 Japanese individuals from the general population and for in vitro functional evaluation.

## Results

### Rare variants of MTTP, PNPLA3, and TM6SF2 in Japanese adults

**Fig. 1:**
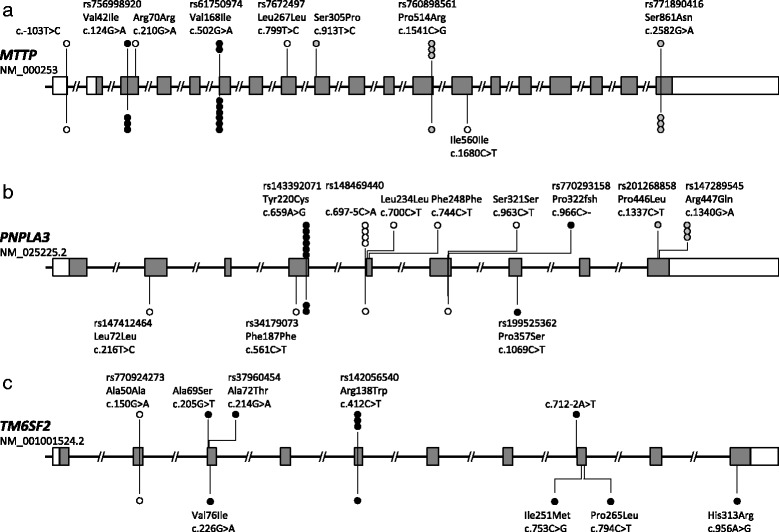
Rare variants discovered after sequencing of 950 Japanese males who did not abuse alcohol. Individual variations (minor allele frequencies less than 2%) are shown according to the genomic position of *MTTP* (**a**), *PNPLA3* (**b**) and *TM6SF2* (**c**). The number of circles on the top and bottom represents the number of times that the variant is observed in cases or controls, respectively. Open circles show synonymous variants and an intronic variant. Gray circles indicate non-synonymous variants that are predicted to be a benign substitution by PolyPhen software. Black circles indicate non-synonymous variants and splicing variants that are predicted to be damaging

Twenty-nine rare (minor allele frequencies <0.02) single-nucleotide variations were identified using GS Junior sequencing in Japanese males in the discovery panel (*n* = 950) who did not abuse alcohol. Coverage of the target sequences was 98%, and the average sequencing depth was 50 ± 36 in 454 reads. All variants were confirmed by direct sequencing (not shown), in which 10 were synonymous, 16 were non-synonymous, two were adjacent to a splicing junction, and one was a frame shift base deletion (Fig. 1). To determine whether the identified rare alleles accumulated in NAFLD cases, burden tests of rare variants in the three genes were evaluated by two mutation sets: (A) non-synonymous, splicing disruption, and frame shift variants, and (B) non-synonymous variants that were expected to be damaging as determined by PolyPhen analysis. In the mutation set (A), rare variants in *PNPLA3* were present significantly more frequently in NAFLD cases (odds ratio (OR) = 3.39, *P* = 0.0409), whereas the association disappeared in the mutation set (B) (Table 1). In contrast, *MTTP* showed no association in set (A) and a marginal negative association in mutation set (B) (OR = 0.3, *P* = 0.0793). *TM6SF2* variants showed no association in either set.

**Table 1:** Association of the burden of rare alleles in MTTP, PNPLA3, and TM6SF2 with the risk of NAFLD in Japanese males

Mutation set			Variant carrier	Non carrier	OR	95% CI	*P*
(A)	*MTTP*	control	13	450	0.55	0.00–1.17	0.269
Non-synonymous		case	8	499			
Splice site	*PNPLA3*	control	3	458	3.39	1.16–12.24	0.0409
Frame shift		case	11	492			
	*TM6SF2*	control	5	458	1.10	0.33–3.62	1
		case	6	501			
(B)	*MTTP*	control	9	453	0.3	0.00–0.90	0.0793
Damaging (PolyPhen)		case	3	504			
Splice site	*PNPLA3*	control	3	459	2.14	0.68–8.33	0.3465
Frame shift		case	7	500			
	*TM6SF2*	control	5	457	1.09	0.40–3.61	1
		case	6	501			

Only SNVs and indels with minor allel frequency less than 1% were considered in burden analysis. Upper mutation set includes missense SNVs, mutation at splice site dinucleotides and frame shift mutatiopns. Damaging (PolyPhen) indicates missense SNVs annoted as ‘possibly damaging’ or ‘probabbly damaging’ by PolyPhen2 HumDiv software. OR (odds ratio) and *P*-values of Fisher’s exact tests are shown

### Genetic associations of the individual rare variants with NALFD and serum lipid levels in Japanese individuals

To investigate whether the identified rare variants were individually associated with NAFLD, non-synonymous rare variants other than singletons, rs148469440 (*PNPLA3* c.697-5C > A), and the wzell-known NAFLD-associated common SNPs (rs738409 and rs58542926) were genotyped using the TaqMan genotyping assay system in 3014 individuals from the Japanese population. The success rate of genotyping was >99.8% in all studied SNPs, and the genotype distributions did not deviate from Hardy-Weinberg equilibrium (Table 2). Logistic regression analysis replicated the significant association of rs738409 (OR = 1.66, 95% confidence interval (CI) = 1.41–1.96, *P* = 1.44E-09) and rs58542926 (OR = 1.8, 95% CI = 1.36–2.38, *P* = 3.39E-05) with NAFLD (Table 2). Among the rare variants typed with TaqMan, rs143392071 (*PNPLA3*, Tyr220Cys) and rs756998920 (*MTTP*, Val42Ile) showed a significant association with ultrasonographic diagnosis of NAFLD (OR = 3.52, 95% CI = 1.38–8.95, *P* = 0.008 for rs143392071 and OR = 0.03, 95% CI = 0.002–0.57, *P* = 0.019 for rs756998920) (Table 2). Although Bonferroni correction (8-fold) decreased the significance, relatively higher ORs for these rare variants were seen compared to common variants (Table 2), suggesting that the associations observed in rs143392071 and rs756998920 were risk and beneficial alleles, respectively, that were independent of the common functional variants for the development of NAFLD.

**Table 2:** Association studies of individual rare alleles with NAFLD in Japanese individuals

Gene	dbSNP ID	Position	Logistic regression analysis			HWE	
			OR	(95% CI)	*P*	MAF	*P*
*MTTP*	rs756998920	Val42Ile	0.03	0.002–0.57	0.019	0.0033	0.855
*MTTP*	rs61750974	Val168Ile	1.28	0.63–2.58	0.493	0.0120	0.396
*MTTP*	rs760898561	Pro514Arg	1.52	0.26–8.73	0.641	0.0018	0.920
*MTTP*	rs771890416	Ser861Asn	0.74	0.07–7.38	0.798	0.0013	0.942
*PNPLA3*	rs143392071	Tyr220Cys	3.52	1.38–8.95	0.008	0.0056	0.755
*PNPLA3*	rs148469440	c.697-5C > A	2.15	0.8–5.47	0.127	0.0047	0.405
*PNPLA3*	rs147289545	Arg447Gln	0.63	0.16–2.4	0.497	0.0032	0.862
*TM6SF2*	rs142056540	Arg138Trp	0.42	0.05–3.65	0.432	0.0010	0.956
*PNPLA3*	rs738409	Ile148Met	1.66	1.41–1.96	1.44E-09	0.4667	0.073
*TM6SF2*	rs58542926	Glu167Lys	1.8	1.36–2.38	3.39E-05	0.0799	0.858

Association of the individual SNP with NAFLD was assessed by Logistic regression analysis, which used age, sex, visceral fat depot, HbA1c, rs738409 and rs58542926 as covariates. Odds ratio (OR), 95% confidence interval (95%CI) and P value are shown. Minor allele frequency (MAF) and P value of Hardy-Weinberg equilibrium (HWE) are also exibited

We next assessed the association of the rare variants with plasma lipid levels. As we previously reported, rs58542926 in *TM6SF2* was significantly associated with plasma triglyceride (TG) levels in Japanese individuals [9]. Therefore, rs58542926 was included as a covariate in multiple regression analysis to assess the genetic involvement of the rare variants in plasma TG or low-density lipoprotein levels. Among the eight rare variants, the minor allele of rs756998920 in *MTTP* significantly decreased the TG level (β = −0.039, *P* = 0.014) (Table 3).

**Table 3:** Association studies of individual rare alleles with plasma lipid levels in Japanese individuals

Gene	dbSNP ID	Position	TG			LDL		
			b	(95% CI)	*P*	b	(95%CI)	*P*
*MTTP*	rs756998920	Val42Ile	−0.039	−0.198 - -0.023	0.014	−0.025	−21.7 - 3.5	0.157
*MTTP*	rs61750974	Val168Ile	−0.022	−0.079 - 0.014	0.167	0.011	−4.4 - 8.7	0.523
*MTTP*	rs760898561	Pro514Arg	0.242	−0.179 - 0.306	0.002	−0.022	−27.8 - 6.1	0.210
*MTTP*	rs771890416	Ser861Asn	−0.006	−0.164 - 0.113	0.242	0.007	−15.9 - 23.8	0.694
*PNPLA3*	rs143392071	Tyr220Cys	0.013	−0.039 - 0.097	0.401	0.020	−4.1 - 15.3	0.259
*PNPLA3*	rs148469440	c.697-5C > A	0.021	−0.023 - 0.121	0.182	−0.019	−15.9 - 4.7	0.288
*PNPLA3*	rs147289545	Arg447Gln	−0.022	−0.155 - 0.026	0.160	−0.012	−17.4 - 8.4	0.492
*TM6SF2*	rs142056540	Arg138Trp	0.011	−0.101 - 0.219	0.471	0.013	−14.5 - 31.3	0.473
*PNPLA3*	rs738409	Ile148Met	−0.025	−0.019 - 0.002	0.121	0.011	−1.0 - 2.0	0.531
*TM6SF2*	rs58542926	Glu167Lys	−0.054	−0.050 - -0.013	0.001	−0.027	−4.5 - 0.7	0.130

Association of the individual SNP with log transformed triglyceride (TG) and LDL-cholesterol (LDL) was assessed by multiple regression model. Age, sex, visceral fat depot, HbA1c and rs58542926 were used as covariates

### Functional validation

**Fig. 2:**
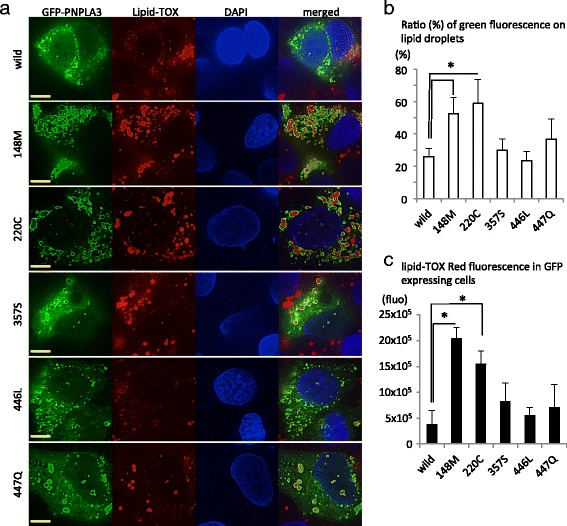
Localization of GFP-tagged PNPLA3 in Huh-7 cells. **a** Huh-7 cells grown on chamber slides were transfected with pEGFP-PNPLA3 encoding each variation labeled on the left. Four hours after the transfection, medium was changed to fresh medium supplemented with 200 μM oleic acid. After incubation for 24 h with oleic acid, Huh-7 cells were fixed and stained with LipidTOX Red and DAPI. The stained cells were visualized under a fluorescence microscope. Yellow scale bars indicate 10 μm. **b** The ratio (%) of GFP-PNPLA3 on lipid droplets to total GFP fluorescence and (**c**) LipidTOX fluorescence intensity in GFP-expressing cells were measured from 10 randomly selected fields. The average value and standard deviations are shown. *P < 0.05 (Student’s t-test)

Enhanced lipid accumulation has been shown in Huh-7 cells expressing 148Met-PNPLA3 [11]. According to this report, influences of the identified non-synonymous rare variants of *PNPLA3* were evaluated by the expression ratio of lipid droplets/cytoplasmic PNPLA3 and by lipid accumulation in PNPLA3-expressing cells. Substitution of Tyr220Cys enhanced the localization of PNPLA3 on lipid droplets and led to enlarged lipid droplets in Huh-7 cells toward levels equivalent to those of Ile148Met. Other variants did not show a significant difference from the wild-type protein (Fig. 2), suggesting that Tyr220Cys actually damages the protein function and that the genetic association with NAFLD is a consequence of this defect.

**Fig. 3:**
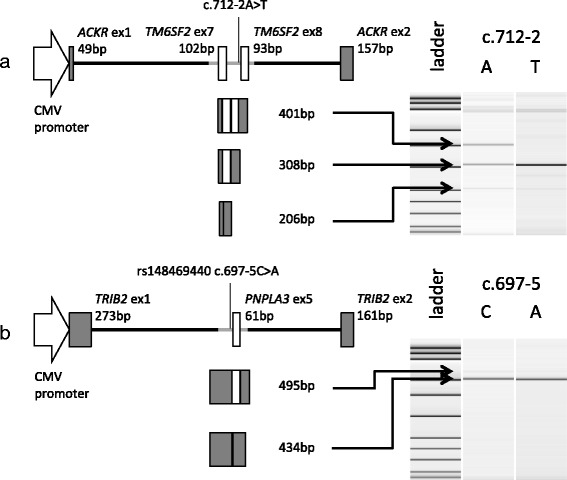
Functional assessment of variants at intron-exon junctions. **a** Intron 6 to intron 8 of *TM6SF2* was inserted into intron 1 of *ACKR1* under control of the CMV promoter. **b** Intron 4 to 5 of *PNPLA3* was inserted into intron 1 of *TRIB2*. The constructs were transfected into Huh-7 cells. Twenty-four hours after transfection, total RNA was extracted from the transfected cells, and the corresponding cDNA was used as a template for RT-PCR. The amplicon sizes of each RT-PCR product were measured with a 2100 BioAnalyzer. Estimated splicing variants from amplicon sizes are shown on the left side

To examine whether the intronic variants of *TM6SF2* (c.712-2A > T) and *PNPLA3* (rs148469440, c.697-5C > A) affect splicing of the adjacent exon, the intron-exon-intron structures were inserted into intron 1 of *atypical chemokine receptor 1* (*ACKR1*) or *tribbles pseudokinase 2* (*TRIB2*), respectively, and the splice variants were detected with RT-PCR (Fig. 3). Substitution of *TM6SF2* (c.712-2A > T) disrupted the splicing acceptor consensus sequence ag > tg and resulted in the skipping of exon 8 (Fig. 3a), which may have led to an in-frame loss of a 31-residue peptide. rs148469440 is localized in the pyrimidine tract of the 3′-splicing sequence, cttgcttgctttgct(c > a)acag, and disrupted joining to *PNPLA3* exon 5 in 80% transcripts (Fig. 3b). The exon skipping of *PNPLA3* exon 5 results in a frame shift, suggesting a deleterious SNP for PNPLA3 function.

**Fig. 4:**
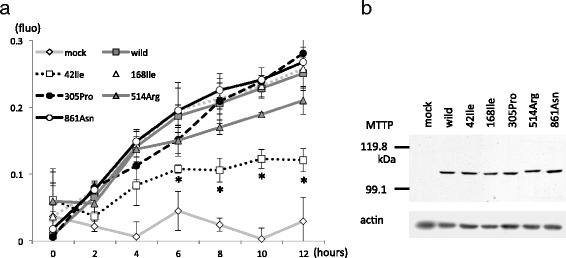
MTP activities of *MTTP* variants. **a** *MTTP* cDNAs encoding the indicated rare variants were inserted into the pCR3 vector. The constructs were transfected into COS-7 cells, and 24 h after the transfection, cells were harvested and homogenized by sonication. Each homogenate containing 100 μg protein was used for triplicate MTP lipid transfer assays. Average fluorescence with standard deviations are shown. Statistical significance compared to wild-type MTTP is indicated. *P < 0.05, Student’s t-test. **b** Remaining homogenate containing 20 μg protein was used for western blotting with anti-MTTP or anti-actin

The functional influences of six non-synonymous rare variants of *MTTP* were assessed in terms of microsomal transfer protein (MTP) activity. Although five amino acid substitutions did not influence the level of protein expressed in COS-7 cells, Val42Ile decreased MTP activity by 47.5% compared to wild-type MTTP (Fig. 4). These results support the genetic association of rs756998920, in which the Val42Ile variant decreases the NAFLD risk and plasma TG (Tables 2 and 3).

## Discussion

Target re-sequencing analysis using next-generation sequencing identified 29 rare variants in 65 Japanese males (6.84%), 12 of which were newly identified base substitutions. Significant accumulation of rare alleles in *PNPLA3* in NAFLD patients and marginal accumulation of rare variants of *MTTP* in non-NAFLD subjects suggested a potential contribution of rare alleles of these genes to the development of NAFLD.

A splicing variant of *TM6SF2* was identified in an NAFLD case and led to deletion of a 31-amino acid peptide. TM6SF2 is an endoplasmic reticulum membranous protein and is predicted to be involved in neutral lipid transfer across the lipid bilayer [10]. The deleted 31 residues are predicted to connect the seventh transmembrane domain to the eighth domain [17] and are conserved from zebrafish to humans. The absence of these 31 residues may destroy the TM6SF2 protein architecture, and the misfolded protein may be degraded and contribute to the development of NAFLD.

Wild-type PNPLA3 has TG hydrolase activity and mediates remodeling of lipid accumulation in hepatocytes. The NAFLD-associated SNP, rs738409, results in an Ile148Met substitution and diminishes the hydrolase activity. The functionally defective PNPLA3 is localized around cytoplasmic lipid droplets and disrupts the access of other lipolytic enzymes, consequently leading to accumulation of cytoplasmic lipid droplets [11, 18]. Among our identified missense variants, Tyr220Cys (rs143392071) showed equivalent lipid accumulation as Ile148Met, and PNPLA3 protein was enriched on lipid droplets in Huh-7 cells. Tyr220 is localized in an alpha-helix structure of a patatin-like domain, which is conserved in many vertebrate species (Additional file 1: Table S2). These results suggest that Tyr220Cys substitution disrupts the hydrolase activity, similar to Ile148Met substitution. Genetic analysis showed that the minor allele of rs143392071 increased the risk of NAFLD. Even with a higher *P* value than rs738409, rs143392071 showed a higher OR for the development of NAFLD (Table 2). Therefore, rs143392071 was considered to be involved in NAFLD susceptibility.

A frame shift variant (Pro322fsh) of *PNPLA3* was identified in an NAFLD case who was homozygous for 148Met. One haploid deletion of 148Met in PNPLA3 was unable to protect the hepatocytes from fat deposits. In addition, minor allele of rs148469440 disrupted regular splicing in 80% of transcripts, which may have decreased PNPLA3 expression but did not decrease NAFLD susceptibility. Recently, a missense SNP, Glu434Lys (rs2294918), was shown to lower the PNPLA3 protein level and protect against steatosis [19]. Our genetic data did not support this observation, but the number of minor allele carriers of the frame shift mutation and rs148469440 was too small to determine the absence of a correlation.

MTTP is a chaperone protein residing in microsomes of hepatocytes and enterocytes and is involved in the assembly of TG-rich ApoB-containing lipoprotein [20]. Homozygotes for the loss-of-function mutation have a very rare disease, abetalipoproteinemia, which is characterized by extremely low plasma lipid levels and hepatic steatosis [21]. However, first-degree relatives are asymptomatic, and the plasma lipid levels and NAFLD susceptibility of heterozygotes for the mutation have not been determined. Although we were unable to identify any apparent loss-of-function mutations among the 950 Japanese males we examined, association studies showed that the rare allele, rs756998920, leads to decreased plasma TG levels and NAFLD risk. In COS-7 cells, MTTP resulting from Val42Ile substitution showed reduced MTP activity. In addition, Val42 is conserved in all vertebrates we studied (Additional file 1: Table S2). MTTP expression in enterocytes affects lipid absorption [21], which potentially regulates energy metabolism and affects NAFLD susceptibility and plasma TG levels. Rubin et al. showed that a common missense SNP, rs3816873 (I128T), is associated with type 2 diabetes [22]. Minor allele carriers of rs756998920 were not susceptible to type 2 diabetes (not shown), but further genetic studies are required to reveal whether heterozygotes for the functionally disrupting allele of *MTTP* have altered plasma TG levels and NAFLD risk.

## Conclusions

In conclusion, the genetically associated and functionally validated SNPs, rs756998920 and rs143392071, were identified by examining 3014 individuals from the Japanese general population, in which 20 and 34 individuals, respectively, were heterozygotes for the allele. Collectively, 1.79% of our study population were estimated carriers of rare variants that were genetically and functionally associated with NAFLD. In addition, a frame shift mutation and a splicing variant (rs148469440) of *PNPLA3* and a splicing mutation of *TM6SF2* were also identified, which were functionally disruptive but not genetically validated. These variant carriers were few in number and not sufficient to explain the missing heritability of NAFLD. Nevertheless, the present study showed that functional variants are still obscure in previously known phenotype-related genes and may contribute to the development of NAFLD to some extent. A limitation of the present study is that the statistical significance was not rigid and disappeared after Bonferroni correction. As previously reported, an extremely large number of samples is required to achieve sufficient statistical power in an exome-wide analysis in the study of rare variants [14]. Further massive population studies or meta-analyses are required to confirm a genetic association for the presented functional rare variants.

## Methods

### Library preparation for target re-sequencing

Males who do not abuse alcohol (less than 20 g consumed/day) and were not carriers of hepatitis B or C virus were chosen for a discovery panel (*n* = 950) from among 3014 Japanese individuals in the general population who underwent a health check-up at Jichi Medical University Hospital (Additional file 1: Table S1). Of these 950 individuals, 458 were diagnosed with non-NAFLD, and 492 were diagnosed with NAFLD by ultrasonography. PCR primers were designed to amplify a total of 34 amplicons covering all the coding regions of the three genes, *PNPLA3*, *MTTP*, and *TM6SF2*. Each sample was amplified with two-step multiplex PCR using the 454 sequencing system as previously described [23]. Forty-eight samples were amplified per sequencing run. In the first step of multiplex PCR, 34 amplicons tailed with M13 sequences were generated in each sample using the KAPA 2G Fast Multiplex PCR Kit (Kapa Biosystems, Boston, MA). For the second nested PCR, the amplicons were further tagged with MID 1 to 50 (except MID 9 and 12) sequences, which were specific for the individual samples, and tailed with 454 GS sequencing adaptors [23]. The amplicon-specific primers with adaptor (M13) sequences are shown in Additional file 1: Table S3. After the second PCR step, individual PCR amplicons were purified with the AMPure XP Kit (Beckman Coulter, Brea, CA) and quantified using the Quant-iT PicoGreen dsDNA Assay Kit (Invitrogen, Eugene, OR). Equivalent amounts of PCR amplicons for the 48 samples per run were mixed into one tube and used as a library for sequencing with a bench-top next-generation sequencer (Roche Diagnostics, Mannheim, Germany). Re-sequenced data for the amplicons were aligned and mapped using CLC genomics workbench software (Version 8, CLC bio Japan) on the *Homo sapiens* (hg19) reference genome data. Variants and mutations in all samples were assembled. Suspected rare variants were confirmed by direct sequencing using a capillary sequencer (Applied Biosystems, Foster City, CA).

### Genotyping

Eight confirmed non-synonymous variants were observed more than two times in the 950 individuals in the discovery panel and were chosen for genotyping in the DNA panel of 3014 Japanese individuals. Simultaneously, rs738409 and rs58542926, which are SNPs that are known to be associated with NAFLD, were included in the study. All SNPs were genotyped with the TaqMan Genotyping Assay Systems (Applied Biosystems).

### Functional evaluation of the rare variants observed in PNPLA3

Five non-synonymous base substitutions in *PNPLA3* (148 M, 220C, 357S, 446 L, and 447Q) were evaluated morphologically for lipid accumulation [11]. The open reading frame of *PNPLA3* (NM_025225) in the pGEM-T vector was edited by the PCR-based mutagenesis procedure. After confirmation of mutagenesis, each open reading frame was ligated into the pEGFP-C1 vector (Clontech, Mountain View, CA). The human hepatoma cell line, Huh-7, was purchased from the JCRB cell bank and maintained in Dulbecco’s modified Eagle’s medium supplemented with 10% fetal calf serum. The pEGFP-*PNPLA3* constructs were transfected into Huh-7 cells grown on chamber slides (AGC Techno Glass, Tokyo, Japan) using lipofectamine 2000 (Life Technologies, Carlsbad, CA). Four hours after the transfection, the medium was changed to fresh medium supplemented with 200 μM oleic acid. After incubation with oleic acid for 24 h, Huh-7 cells were fixed in 3% formaldehyde and stained with LipidTOX Red (Invitrogen) and DAPI. The stained cells were visualized under a fluorescence microscope, and the ratios of GFP-PNPLA3 on lipid droplets to total GFP fluorescence and LipidTOX fluorescence in GFP-expressing cells were measured from 10 randomly selected fields using the associated software (KEYENCE Japan, Osaka, Japan).

Rare variants at exon-intron boundaries were evaluated for splicing efficiency by constructing mini-genes. The PCR fragment of introns 6–8 of *TM6SF2* from the c.712-2A > T mutation carrier and introns 4–5 of *PNPLA3* from the rs148469440 variant carrier were inserted into intron 1 of *ACKR1* and *TRIB2*, respectively. After validation of the construct sequences, the mini-genes were inserted under control of the CMV promoter into the pCR3 vector and transfected into Huh-7 cells. Twenty-four hours after transfection, total RNA was extracted from the transfected cells, and the corresponding cDNA was used as a template for RT-PCR. The amplicon sizes of RT-PCR products were measured by a 2100 BioAnalyzer (Agilent Technologies, Santa Clara, CA).

MTP activity was measured using a fluorometric assay kit (Sigma-Aldrich, St. Louis, MO) according to the manufacturer’s instructions. Open reading frames of *MTTP* cDNA encoding wild type, 42Ile, 149Ile, 305Pro, 514Arg, or 861Asn were inserted into the pCR3 vector and transfected into COS-7 cells using lipofectamine 2000. Twenty-four hours after the transfection, cells were harvested in 400 μL buffer containing protease inhibitors and homogenized by sonication. Each homogenate containing 100 μg protein was used for MTP lipid transfer assays in triplicate. The remaining homogenate containing 20 μg protein was used for western blotting to validate the expression of MTTP protein in COS-7 cells using anti-human MTTP antibody (Flarebio Biotech LLC, College Park, MD).

### Statistical analysis

Logistic regression analysis was applied to assess the rare variants according to the diagnosis of NAFLD and was adjusted for age, sex, daily alcohol consumption, hemoglobin A1c, visceral fat accumulation, rs738409, and rs58542926. All statistical analyses were calculated with SPSS software. The predicted possible damaging impact of non-synonymous SNPs was scored with the Polymorphism Phenotyping v2 (PolyPhen-2) software tool [24].

## Additional file

Additional file 1:
**Table S1.** Metabolic parameters of studied Japanese subjects. **Table S2**. Evolutional conservation of PNPLA3 and MTTP focusing on non-synonymous variants. **Table S3**. Sequences of forward and reverse primers of thirty-four amplicons covering the coding regions of the *PNPLA3*, *MTTP* and *TM6SF2*. (DOCX 34 kb)

## Abbreviations

ACKR1: Atypical chemokine receptor 1
CI: Confidence interval
GCKR: Glucokinase regulator
GWAS: Genome-wide association studies
LYPLAL1: Lysophospholipase like 1
MTP: Microsomal transfer protein
MTTP: Microsomal triglyceride transfer protein
NAFLD: Non-alcoholic fatty liver disease
NCAN-CILP2: Neurocan-cartilage intermediate layer protein 2
OR: Odds ratio
PNPLA3: Patatin like phospholipase domain containing 3
SNP: Single-nucleotide polymorphism
TG: Triglyceride
TM6SF2: Transmembrane 6 superfamily member 2
TRIB2: Tribbles pseudokinase 2
