# Supplementary material for: Identification of deleterious rare variants in MTTP, PNPLA3, and TM6SF2 in Japanese males and association studies with NAFLD
Source: Lipids Health Dis. 2017 Sep 26;16:183. doi: 10.1186/s12944-017-0570-y (PMC5615465; doi:10.1186/s12944-017-0570-y)
Supplement: Additional file 1: — Table S1. Metabolic parameters of studied Japanese subjects. Table S2. Evolutional conservation of PNPLA3 and MTTP focusing on non-synonymous variants. Table S3. Sequences of forward and reverse primers of thirty-four amplicons covering the coding regions of the PNPLA3, MTTP and TM6SF2. (DOCX 34 kb) [file 12944_2017_570_MOESM1_ESM.docx]

Table S1. Metabolic parameters of studied Japanese subjects.

|  | males | | | females | | |
| --- | --- | --- | --- | --- | --- | --- |
|  | control | fatty liver | P | control | fatty liver | P |
| Total number of individuals | 772 | 852 |  | 1089 | 301 |  |
| Individuals without alcohol abuse | 458 | 492 |  | 1025 | 286 |  |
| Age | 53.2 (10.7) | 52 (8.9) | 0.045 | 50.2 (8.8) | 52.4 (8.1) | 0.001 |
| BMI (kg/m^2^) | 22.6 (2.4) | 25.8 (3.2) | <0.001 | 21.7 (2.6) | 26.2 (4.6) | <0.001 |
| Visceral fat area (cm^2^) | 93.2 (36.7) | 154.7 (65.4) | <0.001 | 59.9 (26.7) | 109.7 (46.1) | <0.001 |
| Waist circumstance (cm) | 83.8 (6.3) | 91.5 (8.3) | <0.001 | 78.6 (7.6) | 89.8 (10.2) | <0.001 |
| Fasting plasma glucose (mg/dl) | 99.2 (14.4) | 108.3 (23.3) | <0.001 | 92.8 (10.5) | 103.3 (17.5) | <0.001 |
| HbA1c | 5.25 (0.56) | 5.51 (0.70) | <0.001 | 5.18 (0.39) | 5.52 (0.61) | <0.001 |
| Triglyceride (log_10_(mg/dl)) | 1.96 (0.20) | 2.11 (0.22) | <0.001 | 1.86 (0.18) | 2.02 (0.21) | <0.001 |
| HDL cholesterol (mg/dl) | 60.4 (14.2) | 52.1 (10.9) | <0.001 | 72.1 (15.4) | 62.1 (14.1) | <0.001 |
| LDL cholesterol (mg/dl) | 121.6 (28.3) | 131.1 (26.5) | <0.001 | 122.4 (29.7) | 133.8 (29.4) | <0.001 |
| Systolic blood pressure (mmHg) | 123 (14.3) | 128.3 (14.1) | <0.001 | 118.1 (13.8) | 126.2 (14.6) | <0.001 |
| Diastolic blood pressure (mmHg) | 77.1 (9.1) | 81.9 (9.7) | <0.001 | 73.9 (8.9) | 79.2 (8.9) | <0.001 |
| % Medication for Diabetes | 20.1 | 26.2 | 0.024 | 27.1 | 32.2 | 0.281 |
| % Medication for Dislipidemia | 5.4 | 7.9 | 0.121 | 13.0 | 16.1 | <0.001 |
| % Medication for Hypertension | 47.1 | 48.1 | 0.746 | 57.3 | 58.0 | 0.814 |
| Alcohol consumption (g/day) | 12.2 (5.47) | 10.3 (5.34) | 0.006 | 5.9 (3.2) | 4.4 (2.9) | 0.009 |

For quantitative traits, mean values of individuals without alcoholic abuse are shown with standard deviation in parentheses. For all-or-non traits, percentages under mediaction are shown. P values of Student’s t-test for quqntitative traits and of qui-square test for all-or-non traits are shown. HDL, high-density lipoprotein; LDL, low-density lipoprotein.

Table S2. Evolutional conservation of PNPLA3 and MTTP focusing on non-synonymous variants.

| Species | PNPLA3 | MTTP |
| --- | --- | --- |
| Human  Mouse  Rat  Rhesus macaque  Bovine  Rabbit  Xenopus tropicalis  Zebrafish | I148M  VDALVCSCF**I**PFYSGLIPP  VDALVCSCF**I**PLFSGLIPP  VDALVCSCF**I**PLFSGLIPP  VDALICSCF**I**PFYSGLIPP  VDALCCSCYVPFICGWIPP  VDALLCSCF**I**PFYSGVIPP  IQALICSAFVPIYCGLIPP  IQALICSCF**I**PVYCGLIPP | V42I  LYKLTYSTE**V**LLDRGKGKL  LYKLTYSTE**V**FLDGGKGKP  LYKLTYSTE**V**FLDGGKGKL  LYKLTYSTE**V**LLDRGKGKL  LYKLTYSTE**V**FLDRGKGNL  LYRLTYATE**V**FVDRSKGKL  TYKYFYTTE**V**LIDRPRGSV  LYRYSYSTE**V**GLNRPTGSP |
| Human  Mouse  Rat  Rhesus macaque  Bovine  Rabbit  Xenopus tropicalis  Zebrafish | Y220C  SLRLCTGNL**Y**LLSRAFVPP  SLRLCTGNLQLLTRALFPS  SLRLCTGNLHLLTRALFPS  SLRLCTGNL**Y**LLSRAFVPP  NMQFCTENL**Y**LMFRSLLML  SLRLCLGNV**Y**LMTRALFPP  SIQFSLGNL**Y**RLTRALFPP  SIQFNLDNA**Y**RLSKALFPP | V168I  QLSSGTTNE**V**DISGNCKVT  QLSSGTTNE**V**DISGDCKVT  QLTSGTTNE**V**DISGDCKVT  QLSSGTTNE**V**DISGNCKVT  QLSSGTTNE**V**DISGDCKVT  QLSSGTSNE**V**DISGNCKVT  QLNPGSVSE**V**DVSGNCKVN  QLKSGKMSEADASGKCLVE |
| Human  Mouse  Rat  Rhesus macaque  Bovine  Rabbit  Xenopus tropicalis  Zebrafish | P357S  IRIMSYVML**P**CTLPVESAI  VRILSYIML**P**CSLPVESAI  IRILSYILL**P**CTLPVESAI  IRIMSYVML**P**CTLPVESAI  VKIMSYVML**P**CTLPVESAI  VRVVSYAML**P**CTLPVESAI  MRVASYMVM**P**YTLPVESVV  VRVASYMLL**P**YTLPVESAY | S305P  SHCKGCPSL**S**ELWRSTRKY  RVCKGCPSLAEHWKSIRKN  SVCKGCPSLAEHWQSIRKH  SHCKGCPSILEHWQSTRKY  SKCKGCPSL**S**EHWQSIRKH  SECKGCLSL**S**EHWQSIRSH  SDCKSCPSLLQHWQVNRKH  PKCKGCPNLMETWKAVRSQ |
| Human  Mouse  Rat  Rhesus macaque  Bovine  Rabbit  Xenopus tropicalis  Zebrafish | P446L  PAETKAEAT**P**RSILRSSLN  PGEAKAEGTL-FHPQSSLT  PGEAIAEGTL-FHPKSSLT  PAEAKAEAT**P**RSILRSSLN  PA-----------------  PVGTEAGAAG-SVLGSSZA  -------------------  ------------------- | P514R  TTALQRYDL**P**FITDEVKKT  TTVLQRYDVSFITDEVKKT  TTVLQRYDASFITDEVKKT  TTTLQRYDL**P**FITDEVKKT  ATTLQRYDV**P**FITDEVKKT  TTTLQRYDV**P**FITDEVKKT  ISALQKYDTTFINDEVKKA  ITALQRYDPALITAEVKKA |
| Human  Mouse  Rat  Rhesus macaque  Bovine  Rabbit  Xenopus tropicalis  Zebrafish | R447Q  AETKAEATP**R**SILRSSLNF  GEAKAEGTL-FHPQSSLTF  GEAIAEGTL-FHPKSSLTF  AEAKAEATP**R**SILRSSLNF  A------------------  VGTEAGAAG-SVLGSSZA-  -------------------  ------------------- | S861N  GYVSQKRKE**S**VLAGCEFPL  GYVSRRRKE**S**LVAGCELPL  GYVSRRRKE**S**LVPGCELPL  GYVSQKRKE**S**VLAGCEFPL  GYISRKRKE**S**LIGGCEFPL  GYVSRKRKE**S**VLAGCEFPL  SYISRKRRVQMVAGSELPL  TFSRKRSRDQVVPGSEFPL |

PNPLA3 and MTTP proteins of eight vertebrate species were aligned using multiz46way (http://hgdownload.cse.ucsc.edu/goldenPath/hg19/multiz46way/README.txt) and the peptide sequences of 19 residues surrounding the variation are shown. Bold letters indicate the residue of major allele.

Table S3. Sequences of forward and reverse primers of thirty-four amplicons covering the coding regions of the *PNPLA3*, *MTTP* and *TM6SF2*.

| **Gene** | **Exon to amplify** | **Forward**  **Reverse** | **Sequence** |
| --- | --- | --- | --- |
| ***TM6SF2*** | exon1 | forward | tgtaaaacgacggccATTGGAGACGAGGGTGTCGAT |
|  | exon1 | reverse | ggaaacagctatgacGAAGGGGACTTCCGGTTAGAG |
|  |  |  |  |
|  | exon2 | forward | tgtaaaacgacggccCTGACTTGGCTTCCCTCTCCA |
|  | exon2 | reverse | ggaaacagctatgacGTTTGATTCAGAGGCCCAGTG |
|  |  |  |  |
|  | exon3-4 | forward | tgtaaaacgacggccGACTGTGGTCATGCAACCTTT |
|  | exon3-4 | reverse | ggaaacagctatgacAGAGAGGCGTCCTGATTTCTC |
|  |  |  |  |
|  | exon5 | forward | tgtaaaacgacggccACTGTTATTTCCCCGTTCAA |
|  | exon5 | reverse | ggaaacagctatgacGTCAAGACCATTCTGGGTCAA |
|  |  |  |  |
|  | exon6 | forward | tgtaaaacgacggccCATGGAAGGCAAATACAGCTC |
|  | exon6 | reverse | ggaaacagctatgacAACAGAGTGAAGAAGCCAGCA |
|  |  |  |  |
|  | exon7-8 | forward | tgtaaaacgacggccGGAGGAGCCTTAGGAACCTTT |
|  | exon7-8 | reverse | ggaaacagctatgacTCCCCTCTCACCTGCACCTTA |
|  |  |  |  |
|  | exon9 | forward | tgtaaaacgacggccAAGGGAAATAGAGGAGCAC |
|  | exon9 | reverse | ggaaacagctatgacTAGGGAATCCTGGGATATTGG |
|  |  |  |  |
|  | exon10 | forward | tgtaaaacgacggccGATGGACTGGAGATGGGAAAC |
|  | exon10 | reverse | ggaaacagctatgacCTGCACCCTGAAACACTTAGG |
|  |  |  |  |
| ***PNPLA3*** | exon1 | forward | tgtaaaacgacggccATGCAGTGTCTGCTGGAGTTT |
|  | exon1 | reverse | ggaaacagctatgacGACACTGAGGCAGGGTAGAGA |
|  |  |  |  |
|  | exon2 | forward | tgtaaaacgacggccGCATCCTTGCTGTCTGGTTTC |
|  | exon2 | reverse | ggaaacagctatgacTGACAACGTCCAGATAAGCAA |
|  |  |  |  |
|  | exon3 | forward | tgtaaaacgacggccTAGTTCCCCGTTCTTTTGACC |
|  | exon3 | reverse | ggaaacagctatgacCCAGCTGTGGCTACTCTGTCT |
|  |  |  |  |
|  | exon4 | forward | tgtaaaacgacggccCCTGTTTGTGGCTCTGAGAAG |
|  | exon4 | reverse | ggaaacagctatgacTCCGTTAACCATCAAGGACAG |
|  |  |  |  |
|  | exon5 | forward | tgtaaaacgacggccTAAATGGTGCTTGGTGTCTCC |
|  | exon5 | reverse | ggaaacagctatgacCTTGAAGGCAGAAATGTGCTC |
|  |  |  |  |
|  | exon6 | forward | tgtaaaacgacggccGTTCTCTTCCCTTCCCCTCTT |
|  | exon6 | reverse | ggaaacagctatgacGTGCCATGTTAGGGAGACAGA |
|  |  |  |  |
|  | exon7 | forward | tgtaaaacgacggccCTTTTGTGAGTGCCTCTGACC |
|  | exon7 | reverse | ggaaacagctatgacGGCATTGCACATAGACTGTGA |
|  |  |  |  |
|  | exon8 | forward | tgtaaaacgacggccTGGGAAGTCATCAGATTGGAG |
|  | exon8 | reverse | ggaaacagctatgacCATCAATGCTGCACTCAAAGA |
|  |  |  |  |
|  | exon9 | forward | tgtaaaacgacggccCAGCACTGAGGGCTAGAAGAA |
|  | exon9 | reverse | ggaaacagctatgacCAGGGGTCACTACACAGCAAT |
|  |  |  |  |
|  | exon1 | forward | tgtaaaacgacggccCGTCATTTCCTTCAGCAAGAG |
|  | exon1 | reverse | ggaaacagctatgacGGAGCCTTTCTTCGTTGTTTT |
| ***MTTP*** |  |  |  |
|  | exon2 | forward | tgtaaaacgacggccGTGAACTTAGGTCCTGATTTTGG |
|  | exon2 | reverse | ggaaacagctatgacTTTCAACTCAGCCATGCTTTC |
|  |  |  |  |
|  | exon3 | forward | tgtaaaacgacggccTCAGCAGATGAAGTAGCACCA |
|  | exon3 | reverse | ggaaacagctatgacAAACGGGAACCCAATACAAGT |
|  |  |  |  |
|  | exon4 | forward | tgtaaaacgacggccTTGTGGCCAACTCTTTCTGTT |
|  | exon4 | reverse | ggaaacagctatgacGAACTCAGGGCATTGATTCAG |
|  |  |  |  |
|  | exon5 | forward | tgtaaaacgacggccGAGCCTTCATTATCGGTCTCC |
|  | exon5 | reverse | ggaaacagctatgacTTCATAGGCAAATGCGACTTC |
|  |  |  |  |
|  | exon6-7 | forward | tgtaaaacgacggccGGAGGGAATCAATGAGCTACC |
|  | exon6-7 | reverse | ggaaacagctatgacTTCCCCTTAATGGTTTGTAGGA |
|  |  |  |  |
|  | exon8 | forward | tgtaaaacgacggccTTGTTGCTCCAGAAAGACTTCA |
|  | exon8 | reverse | ggaaacagctatgacGCTGTCATCACAACTCTGTGG |
|  |  |  |  |
|  | exon9 | forward | tgtaaaacgacggccAGTCCCAGGGGTATGATATGG |
|  | exon9 | reverse | ggaaacagctatgacAGTCCCTGCTGAAAATCTGCT |
|  |  |  |  |
|  | exon10 | forward | tgtaaaacgacggccATAAAATGGGGAGGGGTCTTT |
|  | exon10 | reverse | ggaaacagctatgacGTCTCTAGTTGATAGCCTTCATTCAG |
|  |  |  |  |
|  | exon11 | forward | tgtaaaacgacggccAAGTGATGAGGCCCCTTTTTA |
|  | exon11 | reverse | ggaaacagctatgacAGGCAGCATGGTGAGTATCAA |
|  |  |  |  |
|  | exon12 | forward | tgtaaaacgacggccGTCAAGCAACCAATGCAAAAC |
|  | exon12 | reverse | ggaaacagctatgacGAGTGACTTGAGCGGAATTTG |
|  |  |  |  |
|  | exon13 | forward | tgtaaaacgacggccCCTGCTATTCCTGCTGAAATGT |
|  | exon13 | reverse | ggaaacagctatgacCCCAGAGTTACCAGTCATGGA |
|  |  |  |  |
|  | exon14-15 | forward | tgtaaaacgacggccGCTAGCCCTAATCCTGATGCT |
|  | exon14-15 | reverse | ggaaacagctatgacTGGGCAAAATATGACAGAAGC |
|  |  |  |  |
|  | exon16 | forward | tgtaaaacgacggccCAGCTCAGAAGCTTCACCATT |
|  | exon16 | reverse | ggaaacagctatgacCAACATTTTCTCTTTGCCTCCA |
|  |  |  |  |
|  | exon17 | forward | tgtaaaacgacggccGACAGCATGTTTCCAAGCCTA |
|  | exon17 | reverse | ggaaacagctatgacCACAGGGCATCCTGTCTATGT |
|  |  |  |  |
|  | exon18 | forward | tgtaaaacgacggccTCACTGGCATCATACGTTCAG |
|  | exon18 | reverse | ggaaacagctatgacGGCCTTTCTTTCCTGGATAAAT |
|  |  |  |  |
|  | exon19 | forward | tgtaaaacgacggccCCATTCATGGAGTAGCCTTTG |
|  | exon19 | reverse | ggaaacagctatgacGCTGTGGGTAGCATACTGCAT |
|  |  | | |

Lower case letters indicate the M13 sequences.
